# Synergistic Effect of Charge Separation and Multiple Reactive Oxygen Species Generation on Boosting Photocatalytic Degradation of Fluvastatin by ZnIn_2_S_4_/Bi_2_WO_6_ Z-Scheme Heterostructured Photocatalytst

**DOI:** 10.3390/toxics10100555

**Published:** 2022-09-22

**Authors:** Tingting Liu, Fanyu Yang, Liming Wang, Liang Pei, Yushan Hu, Ru Li, Kang Hou, Tianlong Ren

**Affiliations:** 1 School of Environmental and Chemical Engineering, Xi’an Polytechnic University, Xi’an 710048, China; 2 Xinjiang Institute of Ecology and Geography, Chinese Academy of Sciences, Urumqi 830011, China; 3 Institute of Geographic Sciences and Natural Resources Research, Chinese Academy of Sciences, Beijin 100101, China; 4 Northwest Branch of Beijing CCI Architectural Design Co., Ltd., Xi’an 710065, China; 5 Xi’an Capital Water Company Limited, Xi’an 710086, China

**Keywords:** photocatalysis, direct Z-scheme heterojunction, charge separation, redox ability, photoelectrochemical property

## Abstract

The application of semiconductor photocatalysts with narrow band gaps is hindered by the rapid recombination of electron–hole pairs and limitation of multiple reactive oxygen species (ROS) synchronous generation. A n–n-type direct Z-scheme heterostructured photocatalyst was constructed based on the staggered band alignment of bismuth tungstate (Bi_2_WO_6_) and indium zinc sulfide (ZnIn_2_S_4_) to reveal the synergistic effect of charge separation and multiple ROS synchronous generation on boosting photocatalytic performance. Under irradiation, electrons in the conduction band (CB) of Bi_2_WO_6_ and holes in the valence band (VB) of ZnIn_2_S_4_ recombined at interface to prolong the lifetime of electrons in the CB of ZnIn_2_S_4_ and holes in the VB of Bi_2_WO_6_. Meanwhile, the multiple ROS synchronously generated to oxidize pollutant due to the strong redox ability of electrons of ZnIn_2_S_4_ and holes of Bi_2_WO_6_, which was determined by the CB potential of ZnIn_2_S_4_ and VB potential of Bi_2_WO_6_. The results provided valuable insights for the application of photocatalysts with a narrow band gap in the field of water pollution control.

## 1. Introduction

Semiconductor photocatalytic technology converts light energy into chemical energy to oxidize and decompose organic pollutants, which is a sustainable technology to alleviate energy crises and environmental pollution [1,2]. When the photocatalyst is irradiated by incident light with energy equal to or larger than the band gap energy, the electrons in the valence band (VB) absorb photon energy and transit to the conduction band (CB) to generate electron (e^−^)-hole (h^+^) pairs (namely charge carriers). On the one hand, organic pollutants are directly degraded by e^−^ and h^+^ [3]. On the other hand, the organic pollutants are oxidized by reactive oxygen species (ROS) generated through redox reaction by charge carriers, which is the main process for eliminating the harm of organic pollutants to the environment in the photocatalytic system [4,5]. Meanwhile, the recombination of e^−^ and h^+^ occurs through the internal and surface paths to inhibit the generation of ROS, which seriously reduces photocatalytic efficiency. Therefore, excitation of the photocatalyst to produce charge carriers is an important process of photocatalytic reaction, and the redox ability and separation capacity of charge carriers are key factors affecting the photocatalytic efficiency of the system. 

Sunlight is the preferred light source for semiconductor photocatalytic technology, in which ultraviolet light accounts for 7% and visible light for 45.8%. Based on the basic principle of photocatalytic reaction, tuning the band gap to improve the response of visible light with low energy is a hotspot in the field of photocatalysis [6]. A bismuth (Bi)-based photocatalyst has attracted attention due to the advantages of a narrow band gap, low cost, excellent stability and good photocorrosion resistance [7]. The electronic orbital arrangement of bismuth (Bi) is 6s^2^6p^3^, the orbitals of Bi 6s and Bi 6p constituting the VB and CB, respectively, which effectively reduce the band gap to realize the light harvesting of low-energy visible light [8]. The hybridization of the orbitals of Bi 6s and O 2p results in high dispersion of h^+^ in the VB, which contributes to the transition of h^+^ to the photocatalyst surface and inhibition of the charge carriers recombination [9]. In addition, the band gap structure of Bi-based photocatalysts can be tuned to initiate multiple redox reactions by appropriate preparation methods [10].

Although Bi-based photocatalysts show the excellent optical properties of visible light harvesting, the low photocatalytic efficiency still limits its application. Firstly, the VB of a Bi-based photocatalyst is more positive, which is conducive to the generation of hydroxyl radical (·OH) through oxidation of H_2_O and OH^−^ by h^+^. However, the CB of a Bi-based photocatalyst determines the low redox ability of e^−^ to restrict the reduction of O_2_ and synchronous generation of multiple ROS. Secondly, the photocatalytic efficiency is negatively affected by the rapid recombination of charge carriers caused by the narrow band gap. Therefore, tuning the band gap structure of a Bi-based photocatalyst to enhance the redox ability and separation of charge carriers is the key problem to be solved in the field of engineering application.

The construction of a heterojunction is an effective way to tune the band gap structure of a Bi-based photocatalyst. The significant differences in transfer of charge carriers can be observed in the different types of heterojunction, which leads to different regulation mechanisms on charge carriers’ separation and photocatalytic performance. Guan et al. synthesized a type Ι conventional heterojunction of BiOI/Bi_5_O_7_I to realize the e^−^ and h^+^ accumulation on the band gap of BiOI, which contributed to improve the photocatalytic performance [11]. The effective separation of charge carriers was achieved in the p-n heterojunction constructed by AgI decorated with Bi_24_O_31_Br_10_ [12]. Zhan et al. found that a Z-scheme heterojunction improved photocatalytic performance through inhibition of charge carriers recombination [13]. Choosing a photocatalyst with suitable band gap structure to composite with a Bi-based photocatalyst is very important for the heterojunction construction.

Ternary metal sulfide photocatalysts can be synthesized by hydrothermal methods under mild conditions. The morphology, crystal structure and optical property can be controlled through changing the reaction conditions [14]. However, our previous research confirmed that the photocatalytic efficiency of the ternary metal sulfide photocatalyst was restricted by the low oxidation ability of h^+^ because the VB potential was lower than the potential of ·OH generation [15]. Based on the characteristics of band gap structure, research on the construction heterojunction of a Bi-based photocatalyst and ternary metal sulfide photocatalyst was gradually carried out. The results showed that separation of charge carriers was facilitated to improve the photocatalytic performance for hydrogen production and pollutant degradation in the system with heterostructured Bi_2_WO_6_/ZnIn_2_S_4_ [16,17]. The degradation efficiency of dye by double Z-Scheme heterostructured BiVO_4_@ZnIn_2_S_4_/Bi_2_Sn_2_O_7_ photocatalyst with core-shell structure was 12–63 times higher than that of the single component photocatalyst [18]. However, photocatalytic performance of the heterostructure photocatalyst of a Bi-based photocatalyst and ternary metal sulfide photocatalyst has mainly been investigated in the published literature. Few pieces of research discussed the simultaneous regulation mechanism of heterostructures for the enhancement of redox ability and separation of charge carriers. Meanwhile, the synergistic effect of charge carriers separation and multiple ROS generation on boosting photocatalytic performance of the heterostructured photocatalyst had not been analyzed in depth. Consequently, it is necessary to construct a Bi-based/ternary metal sulfide heterojunction and carry out a comprehensive study to reveal the relationship between heterostructures with simultaneous enhancement of redox ability and charge carrier separation. 

In the present work, bismuth tungstate (Bi_2_WO_6_) and indium zinc sulfide (ZnIn_2_S_4_) were selected as typical representatives of Bi-based photocatalysts and ternary metal sulfide photocatalysts to construct the heterojunction based on the staggered band alignment. The structural characteristics of the heterostructured photocatalyst, such as morphology, specific surface area, pore size distribution, and crystal phase were characterized by scanning electron microscopy (SEM), specific surface area analysis (BET) and X-ray diffraction (XRD). UV-Vis spectrophotometry (UV-Vis DRS) and electrochemical workstation were used to investigate the optical and photoelectrochemical properties of the heterostructured photocatalyst. The generation of ROS was analyzed by in-situ capture experiments, which was combined with pollutant removal to evaluate the photocatalytic performance of the heterostructured photocatalyst. The simultaneous regulation mechanism of the heterostructure for the enhancement of redox ability and separation of charge carriers was analyzed. The synergistic effect of charge carrier separation and multiple ROS generation on boosting photocatalytic performance of the heterostructured photocatalyst based on the staggered band alignment was revealed. The results will provide valuable insights for the performance improvement of photocatalysts with narrow band gaps and their application in the field of water pollution control.

## 2. Materials and Methods

### 2.1. Chemicals

Zinc nitrate hexahydrate (AR, Zn(NO_3_)_2_·6H_2_O), thioacetamide (AR, C_2_H_5_NS), bismuth nitrate pentahydrate (≥99%, Bi(NO_3_)_3_·5H_2_O), sodium tungstate dehydrate (≥99.5%, Na_2_WO_4_·2H_2_O), ammonium oxalate (AR, (NH_4_)_2_C_2_O_4_) and isopropyl alcohol (AR, C_3_H_8_O) were purchased from Tianjin Kemiou Chemical Reagent Co., Ltd. (Tianjin, China). Indium nitrate hydrate (99.99%, InN_3_O_9_·xH_2_O), 4-Hydroxy-TEMPO (98%, TEMPOL, C_9_H_18_NO_2_) and fluvastatin sodium (≥98%, C_24_H_25_FNNaO_4_) were purchased from the Aladdin Industrial Corporation (Shanghai, China). Nafion-perfluorinated resin solution (5 wt.% in a mixture of lower aliphatic alcohols and water, contains 45% water) was purchased from Macklin Co., Ltd. (Shanghai, China). All chemicals were used without further purification.

### 2.2. Synthesis

#### 2.2.1. Synthesis of ZnIn_2_S_4_

A total of 0.375 mmol Zn(NO_3_)_2_·6H_2_O, 0.75 mmol InN_3_O_9_·xH_2_O and excess C_2_H_5_NS (6 mmol) were mixed in 75 mL deionized water to completely dissolve under continuous stirring at room temperature. The mixture was transferred into a 100 mL stainless autoclave (Rui Quan Xing, Xi’an, China) with a Teflon liner. The autoclave was sealed and placed in a vacuum oven (Taiste Instrument Co., Ltd., Tianjin, China) at 80 °C for 6 h of hydrothermal reaction, which was naturally cooled to room temperature. The yellow photocatalyst was obtained by vacuum filtration and washed with DI water for several times to be dried at 60 °C.

#### 2.2.2. Synthesis of Composite Photocatalyst

A total of 0.02 mol Bi(NO_3_)_3_·5H_2_O, Na_2_WO_4_·2H_2_O and a certain amount of ZnIn_2_S_4_ were mixed in 150 mL deionized water and continuously stirred at room temperature until a uniform mixture was obtained. The mixture was transferred into a 200 mL stainless autoclave (Rui Quan Xing, Xi’an, China) with a Teflon liner to prepare composite photocatalysts through hydrothermal reaction for 12 h at 160 °C in a vacuum oven (Taiste Instrument Co., Ltd., Tianjin, China). The obtained sample was filtrated and washed several times to be dried at 60 °C. The samples fabricated with 0.6978 g, 1.3956 g and 2.0934 g of ZnIn_2_S_4_ were recorded as 0.1-Zn/Bi, 0.2-Zn/Bi and 0.3-Zn/Bi, respectively. The single component photocatalyst of ZnIn_2_S_4_ and Bi_2_WO_6_ were investigated as control.

### 2.3. Characterization

The morphology was characterized by the Phenom LE field emission scanning electron microscope (FESEM, Phenom, Amsterdam, Holland). The as-prepared photocatalyst was uniformly dispersed on the sample stage to be sprayed gold and characterized at 15 kV. The crystal phase was analyzed by Ultiman IVX X-ray diffraction (XRD, Hitachi, Tokyo, Japan) with Cu Kα radiation in the scanning range of 5–70° at a speed of 10°/min. The voltage and current were 40 kV and 40 mA, respectively. The specific surface area and the pore diameter distribution were obtained from the V-Sorb 2800P BET analyzer (Gold App, Beijing, China). The as-prepared photocatalyst was measured at 77 K after degassing at 70 °C for 12 h under vacuum. The optical property was measured by U3310 UV-Vis spectrophotometry (UV-Vis DRS, Hitachi, Tokyo, Japan) at a wavelength range of 200–900 nm at speed of 600 nm/min.

### 2.4. Photoeletrochemical Measurement

The photoeletrochemical characterization was carried out on an electrochemical workstation (Chenhua Instrument Co., Ltd., Shanghai, China) in a conventional three-electrode configuration, with Pt as the counter electrode and Ag/AgCl as the reference electrode. A 0.2 mol/L Na_2_SO_4_ aqueous solution was used as an electrolyte. A 500 W Xe lamp served as the light source. The working electrodes were prepared as follows: 10 mg of as-prepared photocatalyst was dispersed in 1 mL ethanol solution, and 50 μL of Nafion ethanol solution was added to form a uniform suspension by ultrasound for 30 min. Next, 150 μL of suspension was coated on the surface of ITO glass and dried at room temperature for photoeletrochemical measurement. The reaction was conducted in a nitrogen stream. Transient photocurrent response was measured at 0.5 V with the irradiation/dark interval of 20 s. Electrochemical impedance spectroscopy (EIS) was measured at an AC voltage magnitude of 5 mV with a frequency range of 10^5^ to 10^−2^ Hz, with the initial potential of 0 V.

### 2.5. Photocatalytic Experiment

The photocatalytic experiment was conducted in a CEL-WLAX photochemical reactor (Education Au-light, Beijing, China) with a 500 W Xe lamp emitting UV-Vis light (170–900 nm) with an average intensity of 230 W/m^2^. Fluvastatin at an initial concentration of 10 mg/L was used to evaluate the photocatalytic performance, and the dosage of photocatalyst was 0.2 g/L in all photocatalytic experiments. The adsorption reaction was carried out under dark conditions to achieve adsorption-desorption equilibrium between photocatalyst and pollutant, and then the photocatalytic reaction was carried out under irradiation. Samples were taken at the same time interval and filtered by 0.22 μm PES filter for concentration measurements. All experiments were repeated at least three times to evaluate reproducibility.

### 2.6. ROS Analysis

#### 2.6.1. In-Situ Capture Experiment

An in-situ capture experiment was carried out to analyze the role of ROS generated during the photocatalytic reaction, in which different scavengers were introduced to capture corresponding ROS to evaluate the pollutant removal compared with condition of in the absence of a scavenger. In this study, isopropanol, TEMPOL and ammonium oxalate were used to capture hydroxyl radicals (·OH), superoxide radicals (·O_2_^−^) and holes (h^+^), respectively. The concentration of isopropyl alcohol was 1 mL/L, and the concentration of ammonium oxalate and TEMPOL was 1 mmol/L. The process of in-situ capture experiment was consistent with photocatalytic experiments in addition to the introduction of a scavenger.

#### 2.6.2. Probe Molecular Transformation

Nitroblue tetrazolium (NBT) was used as a probe molecule for quantitative analysis of ·O_2_^−^. 0.025 mmol/L NBT was mixed with 0.2g/L 0.3-Zn/Bi composite photocatalyst in an aqueous solution to react under irradiation. The ·O_2_^−^ concentration was calculated by monitoring the change in the concentration of NBT on UV-2600 ultraviolet visible spectrophotometer (Unico, Shanghai, China) at a wavelength of 259 nm. 

Terephthalic acid (TA) can be used as a probe molecule for quantitative analysis of ·OH 0.5 mmol/L TA was prepared in 2 mmol/L NaOH solution, which was mixed with 0.2g/L 0.3-Zn/Bi composite photocatalyst to react under irradiation. The ·OH concentration was calculated by monitoring the concentration of 2-hydroxyterephthalic acid (2-HTA) on an F-7000 fluorescence spectrophotometer (Hitachi, Tokyo, Japan) at the excitation wavelength of 315 nm and emission wavelength of 425 nm, respectively.

### 2.7. Analysis Methods

The absorbance of the samples was measured at 234 nm by a UV-2600 UV-Visible spectrophotometer (Unico, Shanghai, China). The concentration of fluvastatin was calculated by the standard curve. The removal efficiency was calculated by Equation (1):

η = 1 − C_t_/C_0_ (1)

where C_t_ is the concentration of fluvastatin at specific time intervals and C_0_ is the initial concentration of fluvastatin.

## 3. Results

### 3.1. Morphology

**Figure 1:**
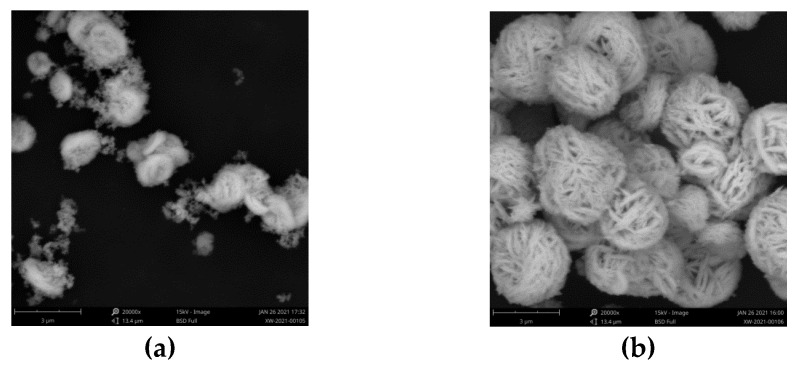

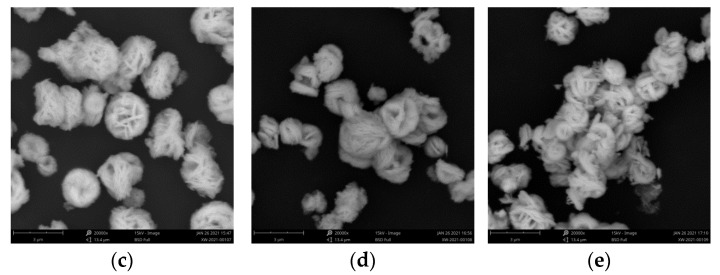
SEM images of as-prepared photocatalyst: (**a**) ZnIn_2_S_4_; (**b**) Bi_2_WO_6_; (**c**) 0.1-Zn/Bi; (**d**) 0.2-Zn/Bi; (**e**) 0.3-Zn/Bi.

The morphology of as-prepared photocatalyst is shown in Figure 1. As shown in Figure 1a, ZnIn_2_S_4_ exhibited oblate morphology with a braided surface of 1–2 μm. In Figure 1b, Bi_2_WO_6_ is a petal-like microsphere assembled by a large number of nanosheets, with an average diameter of approximately 3 μm. It can be seen from Figure 1c–e that the morphology of the composite photocatalyst transited from a petal-like microsphere to a braided microsphere with the increase of the ZnIn_2_S_4_/Bi_2_WO_6_ ratio. The fashioned ZnIn_2_S_4_ was introduced into a hydrothermal reaction to act as the template to induce the assembly of Bi_2_WO_6_ nanosheets on the braided surface, which contributed to fabrication of the composite photocatalyst. The uniform morphology of 0.3-Zn/Bi was obviously observed in Figure 1e compared to the composite photocatalyst with a lower ratio of ZnIn_2_S_4_/Bi_2_WO_6_ (Figure 1c,d), which was beneficial to obtain excellent adsorption and photocatalytic performance.

### 3.2. Crystal Phase

**Figure 2:**
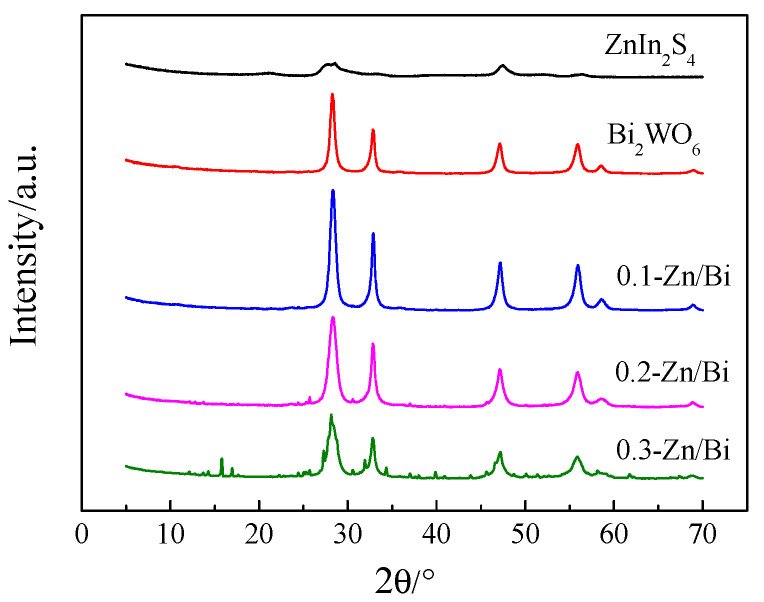
XRD patterns of as-prepared photocatalyst.

The XRD was used to characterize the crystal phase of the as-prepared photocatalysts and the results are shown in Figure 2. In the XRD pattern of ZnIn_2_S_4_, the diffraction peaks of 2θ at 21.5°, 27.6°, 32.4° and 47.2° were assigned to (006), (102), (105) and (110) crystallographic planes, which indicated the formation of pure hexagonal ZnIn_2_S_4_ [19]. For the XRD pattern of Bi_2_WO_6_, the obvious diffraction peaks at 28.3°, 32.9°, 47.1°, 56.0°, 58.5° and 68.8° were assigned to (131), (200), (202), (133), (262) and (400) crystallographic planes, which were consistent with the standard card JCPDS-01-033-1126 and declared the orthogonal phase [20].

As shown in Figure 2, the composite photocatalyst exhibited an orthogonal phase because the diffraction peaks of a composite photocatalyst with a different ZnIn_2_S_4_/Bi_2_WO_6_ ratio were same with that of Bi_2a_WO_6_, indicating that the crystal phase of Bi_2_WO_6_ was unchanged by the introduction of ZnIn_2_S_4_ into the hydrothermal reaction. Moreover, the characteristic diffraction peaks of ZnIn_2_S_4_ was absent in the XRD patterns of the composite photocatalyst, which may be caused by a low ratio of ZnIn_2_S_4_/Bi_2_WO_6_. Meanwhile, the diffraction peak intensity of the composite photocatalyst obviously decreased with the increase of ZnIn_2_S_4_/Bi_2_WO_6_ ratio. In the XRD pattern of 0.3-Zn/Bi, some new diffraction peaks with low intensities were observed. The results showed that the assembly of Bi_2_WO_6_ was affected by ZnIn_2_S_4_ during the hydrothermal reaction, which further confirmed that ZnIn_2_S_4_ and Bi_2_WO_6_ were successfully compounded to form the composite photocatalyst in a hydrothermal reaction. The narrow and sharp diffraction peaks represented high crystallinity of the composite photocatalyst compared to the single component photocatalyst.

### 3.3. Specific Surface Area and Pore Size Distribution

The specific surface area and pore size distribution of the as-prepared photocatalyst were analyzed through nitrogen adsorption-desorption isotherms (Figure S1), and the related data is shown in Table 1. The nitrogen adsorption-desorption isotherms of ZnIn_2_S_4_ and Bi_2_WO_6_ were typical type IV isotherms with H3-type hysteresis loops under relatively high pressure, implying the presence of slit-like pores and capillary condensation in the mesoporous [21,22]. The hysteresis loop of ZnIn_2_S_4_ was significantly larger than that of Bi_2_WO_6_, indicating excellent adsorbability of ZnIn_2_S_4_ compared to Bi_2_WO_6_. As shown in Table 1, the BET surface area, pore volume and average pore size of ZnIn_2_S_4_ were 86.88 m^2^/g, 0.27 cm^3^/g and 9.99 nm, respectively. The corresponding data of Bi_2_WO_6_ were 19.64 m^2^/g, 0.51 cm^3^/g and 34.11 nm, respectively.

The type of nitrogen adsorption-desorption isotherms of the composite photocatalyst with different ZnIn_2_S_4_/Bi_2_WO_6_ ratio were consistent with that of the single component photocatalyst. However, the BET surface area of the composite photocatalyst dramatically increased with the increase of ZnIn_2_S_4_/Bi_2_WO_6_ ratio. The BET surface area of the 0.3-Zn/Bi composite photocatalyst reached 37.97 m^2^/g, which was twice that of Bi_2_WO_6_. Combined with the morphology analysis, the BET surface area of the composite photocatalyst was improved by the introduction of ZnIn_2_S_4_ with a porous braided surface. The larger specific surface area represented excellent photocatalytic performance because more active sites were provided for adsorption and photocatalytic reaction. Meanwhile, the porous structure provided pathways for separation and transfer of charge carriers to promote ROS generation, which played an important role in the enhancement of photocatalytic performance [23,24].

### 3.4. Optical Properties

**Figure 3:**
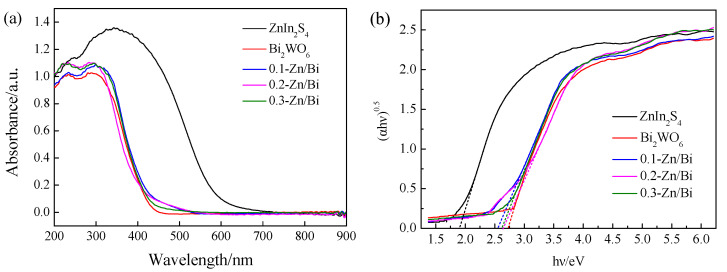
Optical properties of as-prepared photocatalyst: (**a**) UV-Vis diffuse reflectance spectras; (**b**) plots of (α*hν*)^0.5^ versus photon energy *hν*.

The optical properties of a semiconductor photocatalyst is the key factor affecting photocatalytic efficiency. The light harvest of the composite photocatalyst was measured by UV-Vis DRS and the spectras of as-prepared photocatlyst are exhibited in Figure 3a. The strong response to UV-Vis and steep absorption edge at 600 nm of ZnIn_2_S_4_ were confirmed due to inherent narrow band gap. In the spectra of Bi_2_WO_6_, the absorption edge around 450 nm and poor absorptivity of visible light were observed, because only light at short-wavelength with relatively high energy could be absorbed determined by the high band gap energy of Bi_2_WO_6_. The absorption edge of the composite photocatalyst with a different ZnIn_2_S_4_/Bi_2_WO_6_ ratio exhibited slight red shift, demonstrating that enhancement of visible light harvest can be realized by the construction of the composite photocatalyst [25].

The band gap energy of as-prepared photocatalyst can be calculated by Equation (2):

(α*hν*)^n^ = A (*hν* − E_g_) (2)

where α is absorption coefficient in UV-Vis diffuse reflection, *hν* is photon energy, E_g_ is band gap energy, and A is constant. The value of n was 1/2, 2, 3/2 and 3, for allowed direct, allowed indirect, forbidden direct and forbidden indirect transitions, respectively [26]. The band gap energy of as-prepared photocatalyst was determined by the plots of (α*hν*)^1/n^ versus *hν*, in which n was equal to 2 because ZnIn_2_S_4_ and Bi_2_WO_6_ were indirect transition semiconductors [26,27]. From Figure 3b, the band gap energy of ZnIn_2_S_4_ and Bi_2_WO_6_ were 1.88 eV and 2.72 eV, respectively. The band gap energy of the composite photocatalyst slightly decreased compared with Bi_2_WO_6_, but the difference of band gap energy between the composite photocatalyst with different ZnIn_2_S_4_/Bi_2_WO_6_ ratio was negligible. The band gap energy of 0.1-Zn/Bi, 0.2-Zn/Bi and 0.3-Zn/Bi composite photocatalysts were determined to be 2.56 eV, 2.65 eV and 2.62 eV, respectively. The results showed that the introduction of ZnIn_2_S_4_ to composite with Bi_2_WO_6_ could reduce the band gap energy to a certain extent and contribute to improve the utilization rate of light with low energy.

The elemental composition of ZnIn_2_S_4_ and Bi_2_WO_6_ was analyzed by XPS and EDS and the results were displayed in Figure S2. The molar ratio of Zn, ln and S for ZnIn_2_S_4_ was 1:2:3.9, and the molar ratio of Bi, W and O for Bi_2_WO_6_ was 2.3:1:5.8. The CB potential and VB potential of single component photocatalysts were calculated based on Equations (3) and (4) [28]:

E_VB_ = χ − E_e_ + 0.5 E_g_ (3)

E_CB_ = E_VB_ − E_g_ (4)

where χ is the absolute electronegativity of the photocatalyst recorded as the geometric mean value of the absolute electronegativity for constituent atoms; E_e_ is free electron energy (4.5 eV vs. NHE); and E_g_ is band gap energy of the photocatalyst. The calculation results are demonstrated in Table 2.

### 3.5. Photoelectrochemical Property

**Figure 4:**
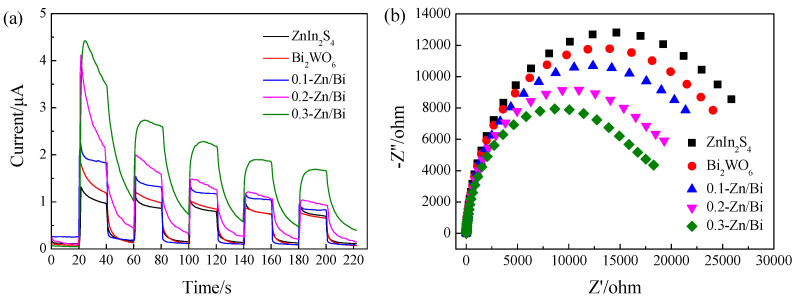
Photoelectrochemical properties of as-prepared photocatalyst: (**a**) Photocurrent-time (I-T) curves; (**b**) EIS plots.

The photocatalyst was excited by light with sufficient energy to generate ROS through the redox reaction of charge carriers. Therefore, the separation and transfer of charge carriers were a crucial process to determine photocatalytic efficiency. The transient photocurrent response and EIS were performed to investigate the separation efficiency of charge carriers. The photocurrent-time (I-T) curves of as-prepared photocatalyst under irradiation for several on-off cycles are displayed in Figure 4a. ZnIn_2_S_4_ and Bi_2_WO_6_ exhibited a very low photocurrent density, which was 1.32 μA and 1.84 μA, respectively. The results demonstrated the fast recombination of charge carriers in a single component photocatalyst, which can be attributed to e^−^ in an unstable state easily returning to the VB to recombine with h^+^ because of narrow band gap structure. Based on the band gap analysis, the band gap of ZnIn_2_S_4_ was obviously more narrow than that of Bi_2_WO_6_, resulting in faster recombination of charge carriers in ZnIn_2_S_4_. Therefore, the photocurrent density of ZnIn_2_S_4_ was lower than that of Bi_2_WO_6_.

The composite photocatalyst showed higher photocurrent response than that of the single component photocatalyst and the photocurrent density increased with the increase of ZnIn_2_S_4_/Bi_2_WO_6_ ratio. The photocurrent density of 0.3-Zn/Bi composite photocatalyst reached 4.42 μA, representing the maximum separation and transfer efficiency of charge carriers. The results revealed more efficient separation and transfer of charge carriers for the composite photocatalyst. The longer lifetime of charge carriers is conducive to ROS generation to improve the photocatalytic efficiency of the composite photocatalyst [30]. According to the results of optical property analysis, the difference of band gap energy between Bi_2_WO_6_ and the composite photocatalyst could be negligible. Therefore, the efficient separation and transfer of charge carriers were attributed to the formation of heterostructure by staggered band alignment of Bi_2_WO6 and ZnIn_2_S_4_. In the heterostructure, the transfer pathway of charge carriers was changed to inhibit the recombination of e^−^ and h^+^, which was conducive to improving the photocatalytic efficiency [31].

EIS is a useful index to characterize transfer of charge carriers through strong correlations between the radius of EIS and transfer performance of charge carriers. The smaller radius of EIS represented smaller resistance, which demonstrated efficient transfer of charge carriers. It could be observed from the EIS of the as-prepared photocatalyst in Figure 4b that the radius of ZnIn_2_S_4_ was larger than that of Bi_2_WO_6_, while the radius of the composite photocatalyst was smaller than that of the single component photocatalyst and the radius decreased with the increase of ZnIn_2_S_4_/Bi_2_WO_6_ ratio. The results further confirmed that the heterostructure was successfully constructed by ZnIn_2_S_4_ and Bi_2_WO_6_, which could promote the separation and transfer of charge carriers to enhance the generation of ROS. The 0.3-Zn/Bi composite photocatalyst showed the highest transfer efficiency of charge carriers, which was in agreement with the results of transient photocurrent response.

### 3.6. Photocatalytic Performance

#### 3.6.1. Removal of Fluvastatin

**Figure 5:**
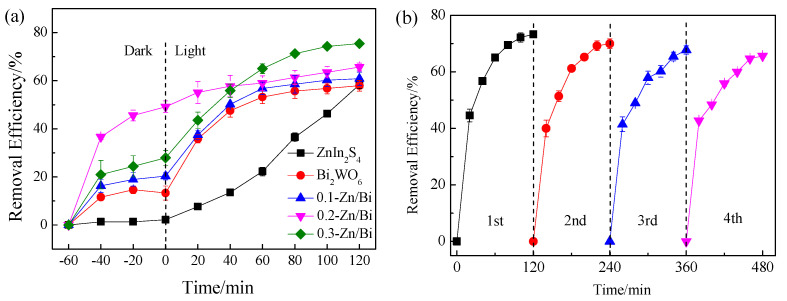
Removal of fluvastatin by as-prepared photocatalyst: (**a**) photocatalytic degradation of fluvastatin; (**b**) the stability of 0.3-Zn/Bi composite photocatalyst for the removal of fluvastatin.

Fluvastatin was selected as a pollutant to investigate the photocatalytic performance of the as-prepared photocatalyst, and the results are demonstrated in Figure 5a. The removal of fluvastatin by ZnIn_2_S_4_ under dark conditions was negligible, indicating poor adsorption performance of ZnIn_2_S_4_ for fluvastatin because of electrostatic repulsion between negatively charged fluvastatin and ZnIn_2_S_4_ [15]. A total of 58.36% of fluvastatin was removed by ZnIn_2_S_4_ under irradiation, indicating that ZnIn_2_S_4_ possessed photocatalytic performance. Fluvastatin could be removed by Bi_2_WO_6_ through adsorption and photocatalytic reaction, with the removal efficiency being 57.89% in an 120 min photocatalytic reaction. In the system with the composite photocatalyst, the removal of fluvastatin by adsorption and photocatalysis was significantly improved compared to the system with the single component photocatalyst. The removal efficiency of fluvastatin increased from 60.84% to 75.47% when the ratio of ZnIn_2_S_4_/Bi_2_WO_6_ increased from 0.1 to 0.3. For the composite photocatalyst, the improvement of adsorption performance was mainly due to the change of morphology and specific surface area by the introduction of ZnIn_2_S_4_, which provided abundant active sites for fluvastatin adsorption. The high adsorption of fluvastatin on 0.2-Zn/Bi was observed in Figure 5a, because of the largest pore volume and average pore size. The photocatalytic performance was enhanced through the construction of the heterostructure to inhibit the recombination of charge carriers and generate multiple ROS.

In conclusion, the photocatalytic performance of the composite photocatalyst was significantly improved and 0.3-Zn/Bi composite photocatalyst exhibited excellent photocatalytic performance.

The stability and lifetime were key factors to evaluate the performance of photocatalysts in engineering applications. The removal of fluvastatin by 0.3-Zn/Bi composite photocatalyst in four cycle experiments was conducted to evaluate the stability of the composite photocatalyst. As shown in Figure 5b, the high removal efficiency of fluvastatin could be observed in the cycle experiments, which remained at 65.61% after four cycles, only decreasing by 7.6% compared to that of the first cycle. The results confirmed that the composite photocatalyst exhibited high stability of photocatalytic performance. The main reasons for the decrease of removal efficiency were the deactivation of photocatalysts caused by active site occupation and leakage of powder photocatalysts in the recovery process. In general, the composite photocatalyst showed high stability of photocatalytic performance, suggesting a possibility for the engineering application.

#### 3.6.2. ROS Analysis

**Figure 6:**
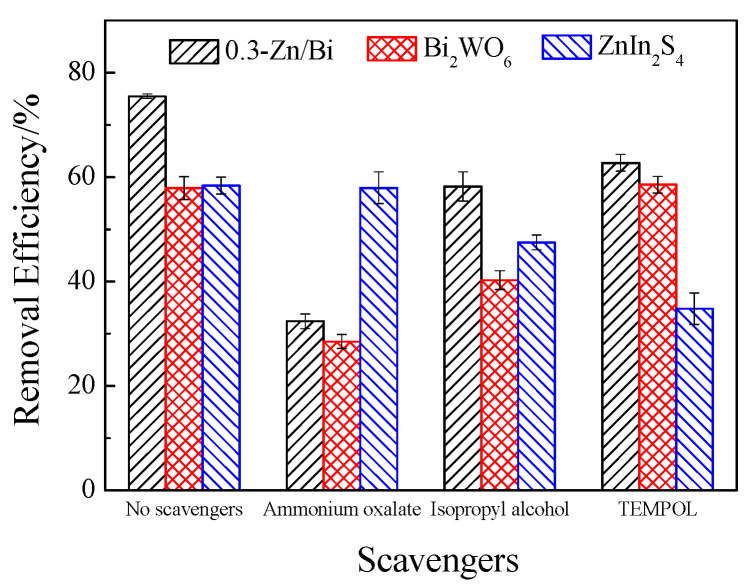
Removal of fluvastatin by as-prepared photocatalyst in the presence of various scavengers: isopropyl alcohol (1ml/L), ammonium oxalate and TEMPOL (1 mmol/L).

The removal efficiency of fluvastatin by the as-prepared photocatalyst in the presence of various scavengers is expressed in Figure 6.

In the photocatalytic system with ZnIn_2_S_4_, the removal efficiency of fluvastatin decreased to 34.81% after the addition of TEMPOL to capture ·O_2_^−^, demonstrating significant inhibition of photocatalytic degradation of fluvastatin compared to the system without a scavenger. When ·OH was captured by isopropyl alcohol, the removal efficiency of fluvastatin also decreased, but was higher than that in the presence of TEMPOL. The effect of the addition of ammonium oxalate to capture h^+^ on photocatalytic degradation of fluvastatin was negligible. The results suggested that fluvastatin was removed by the synergistic oxidation of ·O_2_^−^ and ·OH, in which ·O_2_^−^ was the crucial ROS and ·OH played an auxiliary role. The ROS generation in the photocatalytic system was determined by the potential of CB and VB. The CB potential and VB potential of ZnIn_2_S_4_ were −0.60 eV vs. NHE and +1.28 eV vs. NHE, respectively. O_2_ could be reduced by e^−^ in the CB to generate ·O_2_^−^, but h^+^ in VB could not oxidize OH^−^ and H_2_O to generate ·OH. The production of ·OH relied on a series of reactions associated with ·O_2_^−^, such as when ·O_2_^−^ reacted with e^−^ or H_2_O to produce H_2_O_2_, which further decomposed into ·OH. Therefore, ·O_2_^−^ and ·OH played vital and auxiliary roles in the system with ZnIn_2_S_4_. The ESR analysis in the published literature demonstrated that ·O_2_^−^ and ·OH were generated in the photocatalytic reaction of ZnIn_2_S_4_, but generation of ·OH was inhibited after addition of superoxide dismutase to react with ·O_2_^−^, suggesting that ·OH was generated based on ·O_2_^−^. The results were consistent with the conclusions obtained in the present work [32].

For the system in the presence of Bi_2_WO_6_, the removal efficiency of fluvastatin dramatically decreased to 28.52% and 40.30% when h^+^ and ·OH were captured by ammonium oxalate and isopropyl alcohol, respectively, while the fluvastatin removal did not change after the addition of TEMPOL compared with that of without a scavenger. Fluvastatin could be directly oxidized by h^+^. Meanwhile, OH^−^ and H_2_O were oxidized by h^+^ to generate ·OH, because the VB potential of Bi_2_WO_6_ (+3.16 eV vs. NHE) was more positive than the potential of OH^−^/·OH (+1.99 eV vs. NHE) and H_2_O/·OH (2.27 eV vs. NHE). Fluvastatin was also oxidized by ·OH with strong oxidative ability. However, the reduction of O_2_ by e^−^ to generate ·O_2_^−^ was prevented due to the CB potential of Bi_2_WO_6_ (+0.44 eV vs. NHE) being more positive than the potential of O_2_/·O_2_^−^ (−0.28 eV vs. NHE) [33]. Therefore, h^+^ and ·OH were important ROS for the removal of fluvastatin in the photocatalytic system with Bi_2_WO_6_. According to the results of ROS analysis, the mechanisms of ROS generation in the system with ZnIn_2_S_4_ and Bi_2_WO_6_ were completely opposite because of different band gap structure. However, the staggered band alignment provided an opportunity for the construction of heterostructured photocatalysts to generate multiple ROS.

The ROS generation of the composite photocatalyst was analyzed by 0.3-Zn/Bi composite photocatalyst. The obvious difference of ROS generation between the composite photocatalyst and single component photocatalyst can be observed in Figure 6. The removal efficiency of fluvastatin by 0.3-Zn/Bi composite photocatalyst was 75.47% absence of scavenger, which decreased to 32.39%, 58.22% and 62.73% when h^+^, ·OH and ·O_2_^−^ were captured, respectively. The results demonstrated that the joint oxidation of fluvastatin was achieved by h^+^, ·OH and ·O_2_^−^ in the order of h^+^ > ·OH > ·O_2_^−^.

The concentration of ROS in the system with the composite photocatalyst was quantitatively analyzed by the probe molecular transformation. NBT can react with ·O_2_^−^ to generate insoluble purple product as shown in Equation (5), and the concentration of ·O_2_^−^ in the system can be calculated by the change of the NBT concentration. TA can be used as a probe molecule for quantitative analysis of ·OH because the fluorescent product 2-HTA is generated during the reaction, as shown in Equation (6). The concentration of ·OH in the system can be calculated by the concentration of 2-HTA.

NBT + 4·O_2_^−^ +2H^+^ → formazan + O_2_ (5)

TA + ·OH → 2-HTA(6)

**Figure 7:**
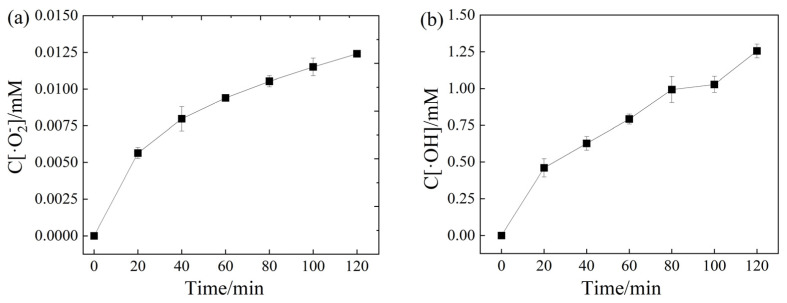
The concentration of ROS generated under irradiation in the presence of 0.3-Zn/Bi composite photocatalyst: (**a**) ·O_2_^−^; (**b**) ·OH.

The concentration of ·OH and ·O_2_^−^ in the system with 0.3-Zn/Bi composite photocatalyst is expressed in Figure 7. The results showed that ROS continuously generated during the reaction because the concentration of ·OH and ·O_2_^−^ increased with the increase of reaction time. After 120min of photocatalytic reaction, the concentration of ·OH and ·O_2_^−^ were 1.26 mmol/L and 2.26 × 10^−4^ mmol/L, respectively. Obviously, ·OH played a more important role than ·O_2_^−^ in the system presence of the composite photocatalyst because the concentration of ·OH was higher than that of ·O_2_^−^. The results were consistent with the conclusions obtained in the in-situ capture experiments.

The goal of the present study was the construction of heterostructured photocatalyst based on staggered band alignment to initiate multiple ROS generation. The results of ROS analysis confirmed that the chain reaction for the generation of ·OH and ·O_2_^−^ could be synchronously initiated due to the CB potential of ZnIn_2_S_4_ and VB potential of Bi_2_WO_6_, which demonstrated that the idea about heterostructure construction was feasible.

### 3.7. Mechanism for the Enhancement of Photocatalytic Performance

**Figure 8:**
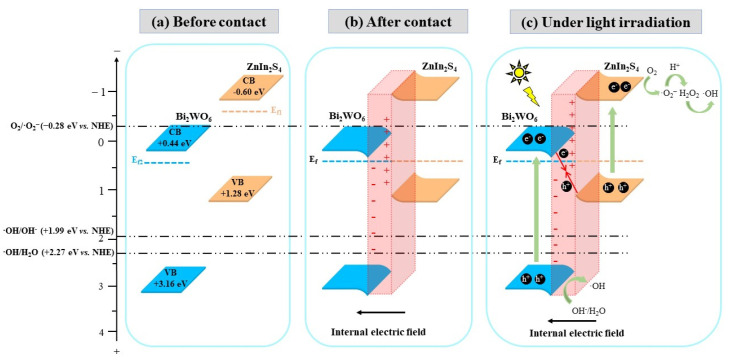
Proposed mechanism for the enhancement of photocatalytic performance of ZnIn_2_S_4_/Bi_2_WO_6_ heterostructured photocatalyst: (**a**) The band structure of the single component photocatalyst before contact; (**b**) The band structure of ZnIn_2_S_4_/Bi_2_WO_6_ heterostructured photocatalyst after contact in hydrothermal reaction; (**c**) The charge carriers transfer pathway of ZnIn_2_S_4_/Bi_2_WO_6_ heterostructured photocatalyst under light irradiation.

In general, photocatalytic performance is determined by the redox ability and lifetime of charge carriers. The key process for the enhancement of photocatalytic performance includes initiating charge carriers, promoting charge carrier separation to prolong its lifetime and inducing the redox reactions of charge carriers. The construction of heterostructured photocatalysts is an effective way to improve the mentioned process. According to the results obtained in the present work, the mechanism for the photocatalytic performance enhancement of ZnIn_2_S_4_/Bi_2_WO_6_ heterostructured photocatalyst was proposed as shown in Figure 8.

ZnIn_2_S_4_ and Bi_2_WO_6_ are n-type semiconductors [34,35]. The potential of CB and VB of ZnIn_2_S_4_ were −0.60 eV vs. NHE and +1.28 eV vs. NHE, and that of Bi_2_WO_6_ were +0.44 eV vs. NHE and +3.16 eV vs. NHE (Figure 8a).

ZnIn_2_S_4_ and Bi_2_WO_6_ were intimately contacted during the hydrothermal reaction to construct an n–n-type heterojunction due to staggered band alignment. The free e^−^ of ZnIn_2_S_4_ with a higher Fermi level (E_f1_) transferred to Bi_2_WO_6_ with a lower Fermi level (E_f2_) through interface to balance their Fermi level. The directional movement of free e^−^ led to positive charge of the ZnIn_2_S_4_ side and negative charge of the Bi_2_WO_6_ side at the interface to establish an internal electric field (IEF). Meanwhile, the band edge of Bi_2_WO_6_ at the interface bent downwards due to accumulation of free e^−^, while the band edge of ZnIn_2_S_4_ at the interface bent upwards due to a decrease of e^−^ density (Figure 8b).

Under light irradiation, e^−^ in the VB of ZnIn_2_S_4_ and Bi_2_WO_6_ absorbed photon energy and transited to the CB to form e^−^ and h^+^ pairs (namely charge carriers). e^−^ in the CB of Bi_2_WO_6_ and h^+^ in the VB of ZnIn_2_S_4_ transferred to interface under IEF and recombined rapidly with each other to form n–n-type direct Z-scheme heterojunctions. The transfer pathway of charge carriers was changed in the heterostructure to prolong the lifetime of e^−^ in the CB of ZnIn_2_S_4_ and h^+^ in the VB of Bi_2_WO_6_, which provided more opportunities for generation of ROS [36]. Meanwhile, the potential barrier caused by band bending prevented e^−^ and h^+^ transferring to opposite directions, ensuring the formation of a direct Z-scheme heterojunction rather than a Type II heterojunction. As the CB potential of ZnIn_2_S_4_ was more negative than the potential of O_2_/·O_2_^−^, O_2_ was reduced by e^−^ to generate ·O_2_^−^. In a similar way, generation of ·OH was achieved through oxidation of OH^−^ and H_2_O by h^+^ in the VB of Bi_2_WO_6_, which potential was more positive than that of OH^−^/·OH and H_2_O/·OH. Differing from the single component photocatalyst, h^+^, ·O_2_^−^ and ·OH were synchronously generated in the system with the heterostructured photocatalyst to synergistically oxidize pollutant, effectively enhancing the photocatalytic performance of the system (Figure 8c).

In summary, the mechanism for the photocatalytic performance enhancement of ZnIn_2_S_4_/Bi_2_WO_6_ heterostructured photocatalyst included two points as follows. Firstly, the well-matched staggered band alignment of ZnIn_2_S_4_ and Bi_2_WO_6_ was utilized to form an n–n direct Z-scheme heterojunction to promote the separation and utilization of charge carriers through changing the transfer pathway of charge carriers. Secondly, the synchronous generation of multiple ROS was achieved to effectively improve the redox ability relying on the advantage of the CB potential of ZnIn_2_S_4_ and VB potential of Bi_2_WO_6_. Therefore, the proposed strategy for the construction of a heterostructured photocatalyst based on the staggered band alignment of a Bi-based photocatalyst and a ternary metal sulfide photocatalyst was a feasible way to obtain an efficient and stable photocatalyst with visible light response.

## 4. Conclusions

In this study, an n–n direct Z-scheme heterostructured photocatalyst was constructed by hydrothermal method based on staggered band alignment of a Bi-based photocatalyst and ternary metal sulfide photocatalyst, whose morphology, crystal phase, specific surface area, pore size distribution, optical property, photoelectrochemical property, photocatalytic performance and ROS generation mechanism were systematically investigated. Moreover, the mechanism for the enhancement of photocatalytic performance was discussed. The main conclusions could be summarized as follows.

The morphology of the heterostructured photocatalyst transited from a petal-like microsphere to a braided microsphere with the increase of the ZnIn_2_S_4_/Bi_2_WO_6_ ratio. The uniform morphology of the heterostructured photocatalyst was conducive to obtain efficient and stable photocatalytic performance. The crystal phase of Bi_2_WO_6_ did not change with the introduction of ZnIn_2_S_4_ into the hydrothermal reaction, and the heterostructured photocatalyst exhibited high crystallinity of the orthogonal phase, which was the same as that of Bi_2_WO_6_. As the ratio of ZnIn_2_S_4_/Bi_2_WO_6_ increased, the BET surface area of the heterostructured photocatalyst increased dramatically. The BET surface area of 0.3-Zn/Bi composite photocatalyst was twice than that of Bi_2_WO_6_, which provided more active sites for adsorption and photocatalytic reaction to improve the photocatalytic performance. The difference of visible light response between the heterostructured photocatalyst and single component photocatalyst was negligible. However, the transient photocurrent response of the heterostructured photocatalyst was significantly improved and the EIS of the heterostructured photocatalyst was obviously decreased, confirming that charge carriers were effectively separated and transferred to prolong its lifetime under irradiation.

The photocatalytic performance of heterostructured photocatalysts was significantly improved, and 75.47% of fluvastatin was removed, by a 0.3-Zn/Bi composite photocatalyst with high stability. In the system with a heterostructured photocatalyst, the joint oxidation of fluvastatin was achieved by multiple ROS, in the order of h^+^ > ·OH > ·O_2_^−^. The mechanism for the photocatalytic performance enhancement of ZnIn_2_S_4_/Bi_2_WO_6_ heterostructured photocatalyst included two points as follows. Firstly, the well-matched staggered-band alignment of ZnIn_2_S_4_ and Bi_2_WO_6_ was utilized to form an n–n direct Z-scheme heterojunction to inhibit recombination of charge carriers and improve the utilization of e^−^ and h^+^ through changing the transfer pathway of charge carriers. Secondly, the synchronous generation of multiple ROS was achieved to effectively improve the redox ability, relying on the advantage of the CB potential of ZnIn_2_S_4_ and VB potential of Bi_2_WO_6_.

In conclusion, the proposed strategy for the construction of a heterostructured photocatalyst based on the staggered band alignment of a Bi-based photocatalyst and ternary metal sulfide photocatalyst was a feasible way to obtain efficient and stable photocatalysts with visible light response. The results provided valuable insights for the performance improvement of photocatalysts with narrow band gaps and their application in the field of water pollution control.

## Figures and Tables

**Table 1:** The specific surface area and pore size distribution of as-prepared photocatalyst.

Photocatalyst	S_BET_ (m^2^/g)	Pore Volume (cm^3^/g)	Average Pore Size (nm)
ZnIn_2_S_4_	86.88	0.27	9.99
Bi_2_WO_6_	19.64	0.51	34.11
0.1-Zn/Bi	32.87	0.19	18.80
0.2-Zn/Bi	37.36	0.75	40.10
0.3-Zn/Bi	37.97	0.50	24.78

**Table 2:** The calculated results of band gap energy and band gap potential of as-prepared photocatalyst.

Photocatalyst	Element	Element Electronegativity (eV) [29]	Molar Ratio	χ (eV)	E_g_ (eV)	E_VB_ (eV)	E_CB_ (eV)
ZnIn_2_S_4_	Zn	4.45	1	4.84	1.88	+1.28	−0.60
	In	3.10	2				
	S	6.22	3.9				
Bi_2_WO_6_	Bi	4.69	2.3	6.30	2.72	+3.16	+0.44
	W	4.40	1				
	O	77.54	5.8				
0.1-Zn/Bi					2.56		
0.2-Zn/Bi					2.65		
0.3-Zn/Bi					2.62		

## Data Availability

Not applicable.

## Supplementary Materials

The following supporting information can be downloaded at: https://www.mdpi.com/article/10.3390/toxics10100555/s1. Figure S1: titleNitrogen adsorption-desorption isotherms of as-prepared photocatalyst: (**a**) ZnIn2S4; (**b**) Bi2WO6; (**c**) 0.1-Zn/Bi; (**d**) 0.2-Zn/Bi; (**e**) 0.3-Zn/Bi. Figure S2: Elemental composition of as-prepared photocatalyst: (**a**) survey XPS spectra of ZnIn_2_S_4_; (**b**) high resolution XPS spectra of Zn 2p; (**c**) high resolution XPS spectra of In 3d; (**d**) high resolution XPS spectra of S 2p; (**e**) EDS of Bi_2_WO_6_.
