# Supplementary material for: Synergistic Effect of Charge Separation and Multiple Reactive Oxygen Species Generation on Boosting Photocatalytic Degradation of Fluvastatin by ZnIn2S4/Bi2WO6 Z-Scheme Heterostructured Photocatalytst
Source: Toxics. 2022 Sep 22;10(10):555. doi: 10.3390/toxics10100555 (PMC9612086; doi:10.3390/toxics10100555)
Supplement: Supplementary file 1 [file toxics-10-00555-s001.zip › toxics-1902607-supplementary.pdf]

toxics

MDPI

Synergistic Effect of Charge Separation and Multiple Reactive Oxygen Species Generation on Boosting Photocatalytic Degradation of Fluvastatin by $ZnIn_2S_4/Bi_2WO_6$ Z-Scheme Heterostructured Photocatalytst

Tingting Liu [1,*], Fanyu Yang [1], Liming Wang [1], Liang Pei [2,3,*], Yushan Hu [4], Ru Li [1], Kang Hou [1] and Tianlong Ren [5]

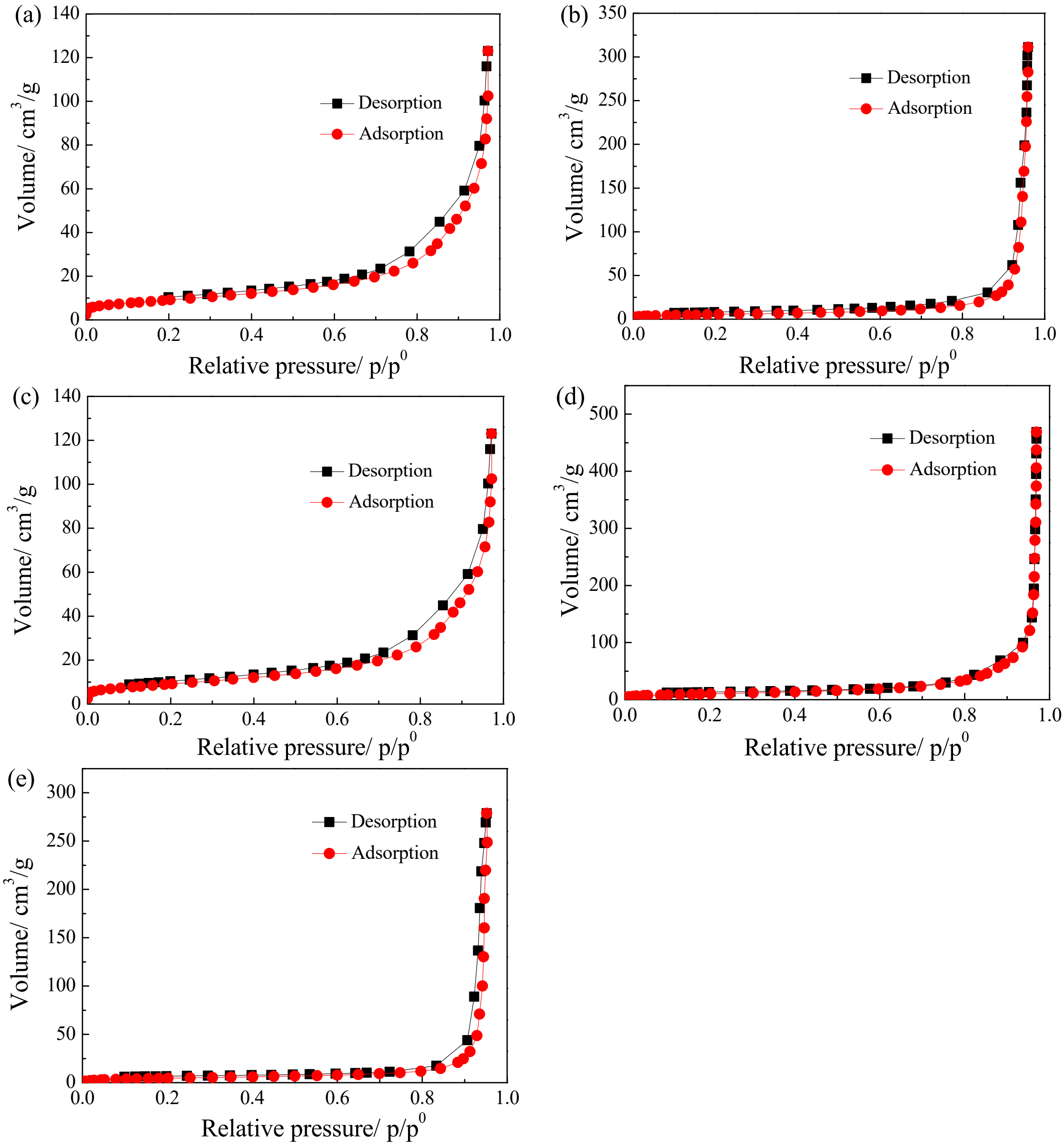

Figure S1. Nitrogen adsorption-desorption isotherms of as-prepared photocatalyst: (**a**) ZnIn2S4; (**b**) Bi2WO6; (**c**) 0.1-Zn/Bi; (**d**) 0.2-Zn/Bi; (**e**) 0.3-Zn/Bi.

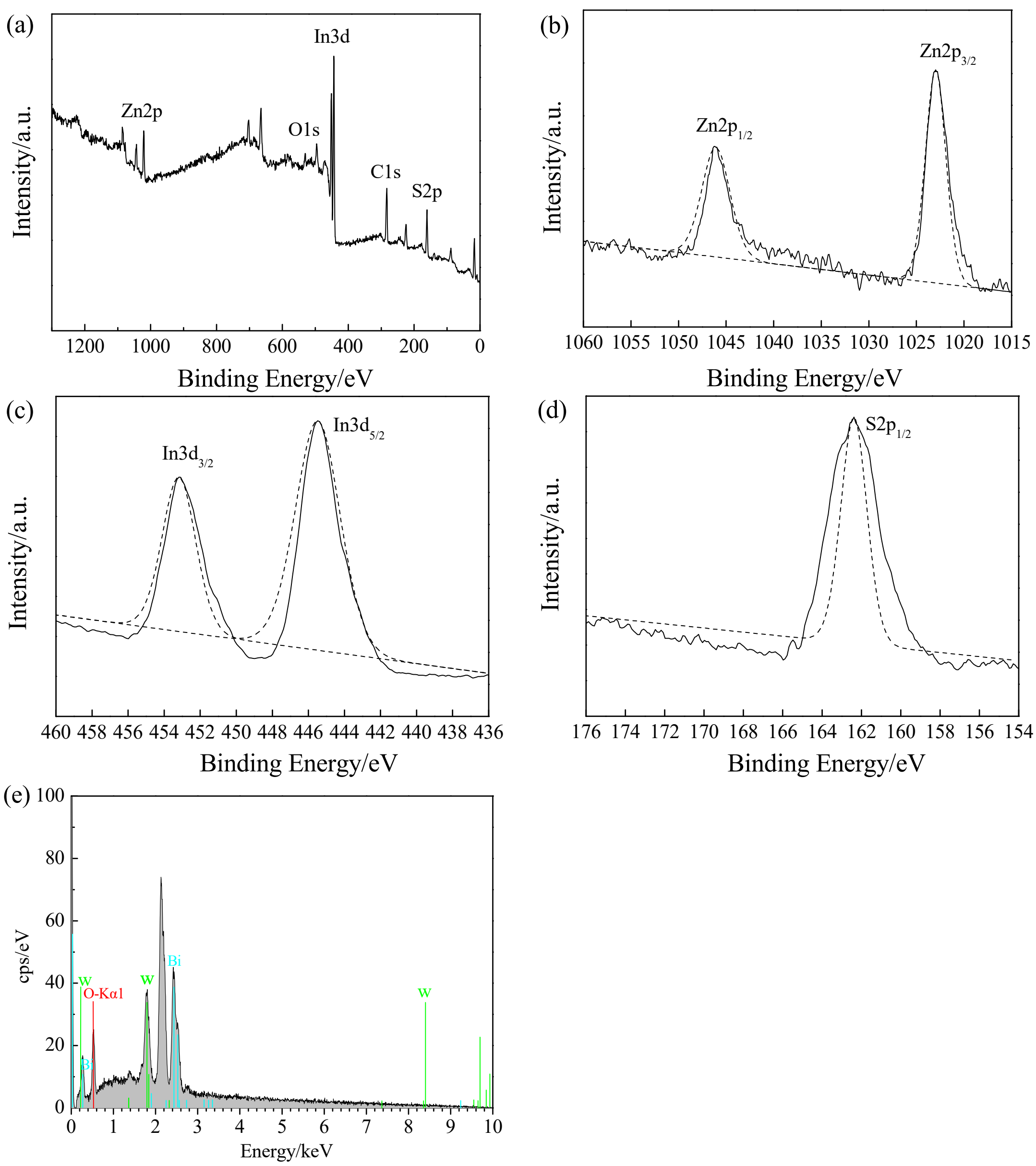

Figure S2. Elemental composition of as-prepared photocatalyst: (**a**) survey XPS spectra of $ZnIn_2S_4$; (**b**) high resolution XPS spectra of Zn 2p; (**c**) high resolution XPS spectra of In 3d; (**d**) high resolution XPS spectra of S 2p; (**e**) EDS of Bi_2WO_6.
